# The three-dimensional finite element model of unilateral complete cleft lip and palate and mechanical analysis of the oral surfaces

**DOI:** 10.1186/s40902-024-00452-7

**Published:** 2025-03-05

**Authors:** Qingqian Wei, Hao Liang, Jingyi Wang, Fei Chen, Yinyue Chen, Yiwei Liu, Haidong Li

**Affiliations:** 1 Plastic Surgery Hospital, Chinese Academy of Medical Sciences and Peking Union Medical College, Beijing； Morin Dawa Daur Autonomous Banner People’s Hospital, Hulunbeir City, Inner Mongolia Autonomous Region, China; 2 Beijing University of Technology, No. 5 Jinyuanzhuang Road, Shijingshan District, Beijing, China; 3 The First Clinical College, Guangzhou Medical University, Guangzhou, China; 4 The Third Clinical College, Guangzhou Medical University, Guangzhou, China; 5 Morin Dawa Daur Autonomous Banner People’s Hospital，Hulunbeir City, Inner Mongolia Autonomous Region； Plastic Surgery Hospital, Chinese Academy of Medical Sciences and Peking Union Medical College, Beijing, China

**Keywords:** Unilateral complete cleft palate, Three-dimensional finite element model analysis, Oral and nasal surface mechanics, CT image

## Abstract

**Background:**

Cleft palate is a prevalent oral and maxillofacial malformation that requires complex surgical interventions. In cleft palate repair, managing flap tension is critical to avoid complications such as flap rupture and impaired healing. Additionally, excessive flap movement can compromise blood supply, affecting postoperative outcomes. A thorough understanding of these biomechanical factors is crucial for surgical success.

**Methods:**

A three-dimensional finite element model was developed using CT scan data to simulate the biomechanical behavior of the cleft palate under surgical conditions. The model was constructed and analyzed using ANSYS Workbench and related software, incorporating material properties of bone, mucosa, and muscle. Stress and deformation distributions were calculated to evaluate surgical incision points and flap movement.

**Results:**

The model identified critical areas of high tension and movement along the surgical incisions on both oral and nasal surfaces. The maximum deformation observed was 3.9885 mm, with stress concentration points along the suture lines and flap edges. The results highlighted specific regions prone to mechanical stress, which are crucial for optimizing surgical strategies.

**Conclusion:**

This study demonstrates the potential of a 3D finite element model in predicting mechanical responses of the cleft palate during surgical repair. The findings provide surgeons with valuable insights for improving incision placement, flap design, and suturing techniques to minimize tension and enhance healing. This personalized approach could significantly improve surgical outcomes and reduce postoperative complications in cleft palate repair.

## Background

Cleft lip and palate are among the most common congenital facial abnormalities, occurring in one out of every 700 newborns [1]. Patients suffer significantly from malformations and pararthria, as well as epistaxis, caused by undeveloped nasal bones, dysfunctional oral swallowing muscles, and multiple dental diseases [2–4]. Unilateral complete cleft palate, a type of cleft where the division extends from one side through both the soft and hard palates, is associated with a higher incidence of upper respiratory tract obstruction and primarily relies on early surgical intervention [5].

Surgical outcomes depend not only on the surgeon’s thorough understanding of the anatomical structure but also on an appropriate operation plan that includes the suitable size and tension of the surgical incision and optimal flap blood supply. Two-flap palatoplasty is commonly used for complete unilateral or bilateral hard clefts in first-stage reconstruction, while Von Langenbeck palatoplasty, when combined with intravelar veloplasty or Furlow double-opposing Z-plasty, can repair complete clefts. Intravelar veloplasty was applied in 94% of soft cleft palate cases. Flaps are increasingly used in these surgeries, playing a crucial role; for instance, 84% of cleft hard palate reconstructions employed the vomer flap [6, 7]. The buccal fat flap has become popular to minimize transverse maxillary growth restriction [8], and the buccinator myomucosal flap has been considered to reduce complications such as fistula formation [9]. Though the surgical techniques above were considered efficient, they may be followed by complications of separate surgeries and secondary operations. Two-flap palatoplasty is relatively simple, suitable for a wide range of cleft palate cases, and can effectively close a large range of cleft. However, it may cause tension in the palate, which may affect postoperative recovery such as oronasal fistula [10, 11]. Although Von Langenbeck palatoplasty is suitable for repairing small widths, especially in the soft palate. For wider cleft palates, it may be less suitable and there is a higher risk of recurrence of the cleft [12]. Furlow Double-opposing Z-plasty is performed on patients with soft palate cleft or speech impairment through two symmetrical “Z” incisions in the soft palate [13]. However, the procedure is long and complicated and may have limited results for severe cleft palates [14]. Based on the conditions above, complications like a failure of pre-existing palatal fistula, velopharyngeal impairment, closure failure of pre-existing alveolar fistula et still occur [15]. When designing operation flaps, incision tension should be carefully managed to prevent postoperative fistula at low tension and wound dehiscence with high tension at wound edges [16]. A novel surgical technique using force balance reconstruction performed satisfying outcomes [17]. To perform a simple-step operation with the optimal force of the orbicularis muscle in the reconstruction of CLP, Huang and his team used finite element (FE) analysis to model the biomechanics of the novel surgical method. The stress of the postoperative muscular system is minimal under different lip shape conditions, with effective prevention of secondary surgery [18].

Compared with the two-dimensional (2D) FE model, which could not perform a reliable quantitative stress analysis [19], the three-dimensional (3D) FE model deprived of computed tomography could better understand the mechanical behavior of multiple complex objections [20]. The main current studies only focused on the comparison of biomechanical stabilities of each surgical operation or single instrument utilization, such as the biomechanical effects of bone grafts [21] or palate expanders [22]. Our study established a 3D FE model comparing tension at common incision sites to identify optimal choices among possible operational management strategies, reducing postoperative complications and achieving perioperative goals.

## Methods

### Geometric model acquisition and grid division

Cleft palate reconstruction operations deal with a uniquely complex, non-geometric structure. The STL model was obtained from the tomography data using 3DSlicer software. Reverse engineering and 3D CAD activities using ANSYS Spaceclaim software to produce solid models suitable for the analysis environment, and optimized grid generation activities using ANSYS Workbench software. To address the inevitable deviations from reality due to sharp points, the PLY file was imported into Geomagic for image preprocessing, which included adding 0.5 mm depth and applying surface smoothing. After surface fitting, an entity file in STP (standard for the exchange of product data) format was generated.

### Material properties and model construction

To accurately reflect the biomechanical properties of the palate, we considered its structural complexity, including interactions among bone, mucosa, and muscle. We employed a linear elastic material model for the soft tissue of the airway wall and muscles. Although this model neglects some nonlinear and viscoelastic properties, it remains sufficiently accurate for transformations less than 2 mm. Therefore, we ultimately selected a linear elastic material with isotropic properties to minimize computational load and maintain accuracy.

Considering the structure of the palate, we choose to build the model based on the biomechanical parameters of the soft tissue of the airway wall and muscles. The Young’s modulus of the soft tissue of the airway wall was 7.59 MPa, Poisson’s ratio was 0.49, bulk modulus was 126.5 MPa, and shear modulus was 2.547 MPa [23]. The Young’s modulus of the muscle is 1 MPa, the Poisson’s ratio is 0.45, the bulk modulus is 3.3333 MPa, and the shear modulus is 0.34483 MPa [24].

**Fig. 1:**
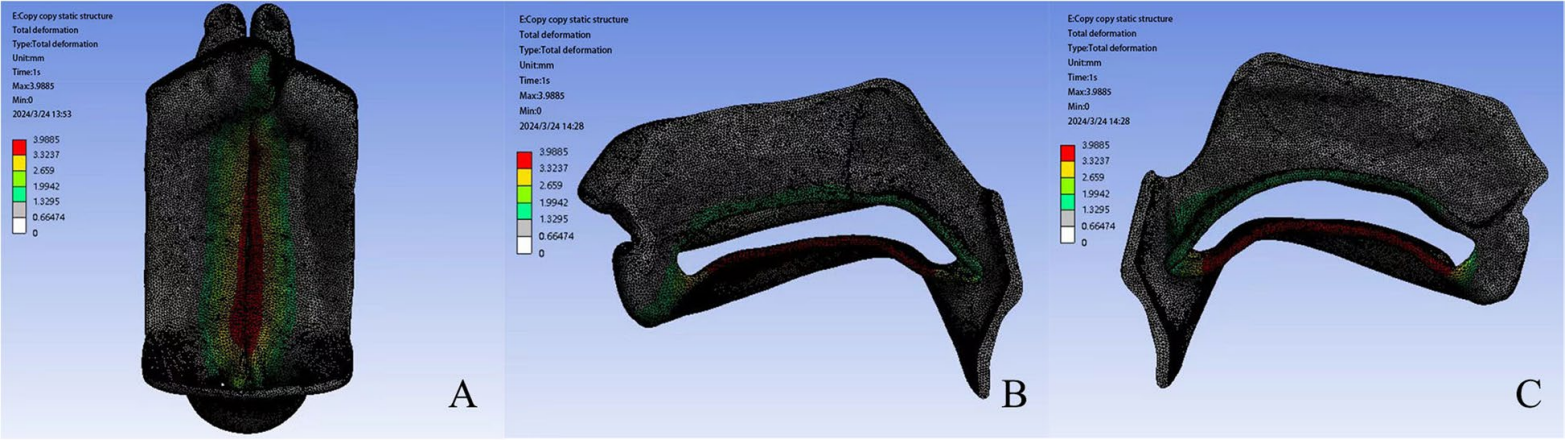
**A** General deformation diagram. **B** Deformable view of the left section. **C** Deformable view of the right section

We chose to build the model on the ANSYS Workbench, which offers tetrahedral and hexahedral mesh types. Due to the rough surface of the medical models, the tetrahedral mesh was selected with a mesh size of 0.15 mm. The mesh generation resulted in a total of 944,475 elements and 472,265 nodes, as shown in Fig. 1A.

### Boundary conditions and contact point definition

For boundary conditions, we established fixed supports around the model’s perimeter and carefully applied displacement to simulate surgical conditions. We identified and marked 12 key points along the flap incision to represent the oral surface, using the “Insert Stress Probe” command in ANSYS Mechanical Enterprise Solver to evaluate stress at these points during simulated surgery. A similar analysis was performed for the nasal surface, facilitating the identification of stress concentrations critical for surgical planning.

### Interaction of tissue structures

The model incorporates different tissue structures, including bone, mucosa, and muscle, by assigning distinct material properties to each component. This multi-material approach allows us to study their interactions under surgical conditions, providing insights into how these elements influence the overall biomechanical response during cleft palate repair.

## Results

### Deformation condition

**Fig. 2:**
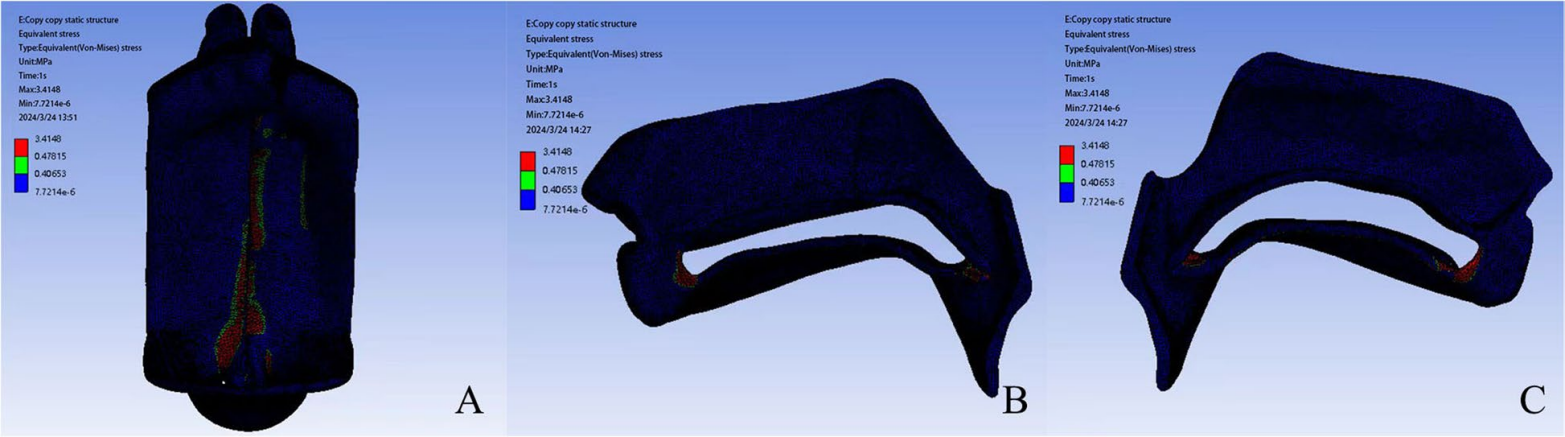
**A** Equal-effect map. **B** Left section stress map. **C** Right section stress map

We successfully simulated the deformation distribution of the cleft palate under realistic conditions. In the model, bright colors indicate areas of higher movement distance/stress (see Figs. 1 and 2). The most significant deformation along the right suture is at node 6 (1.1717 mm), while on the left palate, it is at node 12 (2.0621 mm) (Table 1). Node 6 shows the most notable deformation (3.9571 mm) in the right oral facial surgery. Correspondingly, nodes 10 and 11, both with a deformation of 3.6056 mm, are the most flexible sites in the left oral facial surgery (Table 2). In the nasal operation flaps, node 6 on the right experiences the highest tension (1.6872 MPa), and on the left, it is node 11 (1.6233 MPa) (Table 3). It is noteworthy that the deformations along the oral facial fissure and the nasal operation flap are relatively consistent (Table 4). The observed maximum deformation value is 3.9885 mm (as shown in Fig. 1).

**Table 1:** Strain stress table at each point on the side of the seam, strain unit: mm, stress unit: MPa

	1	2	3	4	5	6	7	8	9	10	11	12
Strain	0.39	0.05	0.79	0.41	0.98	1.17	1.96	0.68	0.24	0.54	1.12	2.06
Stress	0.21	0.06	0.32	0.15	0.15	0.12	0.19	0.08	0.28	0.21	0.22	0.43

**Table 2:** Stress–strain table at each point during postoperative suture of the oral surface side, strain unit: mm, stress unit: Mpa

	1	2	3	4	5	6	7	8	9	10	11	12
Strain	3.16	3.78	3.78	3.88	3.92	3.95	3.31	3.39	3.58	3.60	3.60	3.59
Stress	0.93	0.77	0.17	0.25	0.23	0.25	0.13	0.07	0.19	0.29	0.19	0.12

**Table 3:** The stress and strain table at each point during the suture of the nasal surface, the strain unit: mm, the stress unit: MPa

	1	2	3	4	5	6	7	8	9	10	11	12
Strain	1.30	1.41	1.47	1.36	1.44	1.68	1.47	1.40	1.40	1.41	1.62	1.44
Stress	0.01	0.01	0.03	0.03	0.01	0.08	0.38	0.06	0.04	0.04	0.13	0.07

**Table 4:** Summary of the highest and lowest tension and deformation points across the oral and nasal surfaces

Location	Node	Maximum deformation (mm)	Maximum stress (MPa)
Right suture	6	1.1717	0.32388
Left suture	6	2.0621	0.4379
Right oral surface	6	3.9571	0.93218
Left oral surface	6	3.6056	0.29217
Right nasal surface	6	1.6872	0.080944
Left nasal surface	6	1.6233	0.38239

### Stress condition

**Fig. 3:**
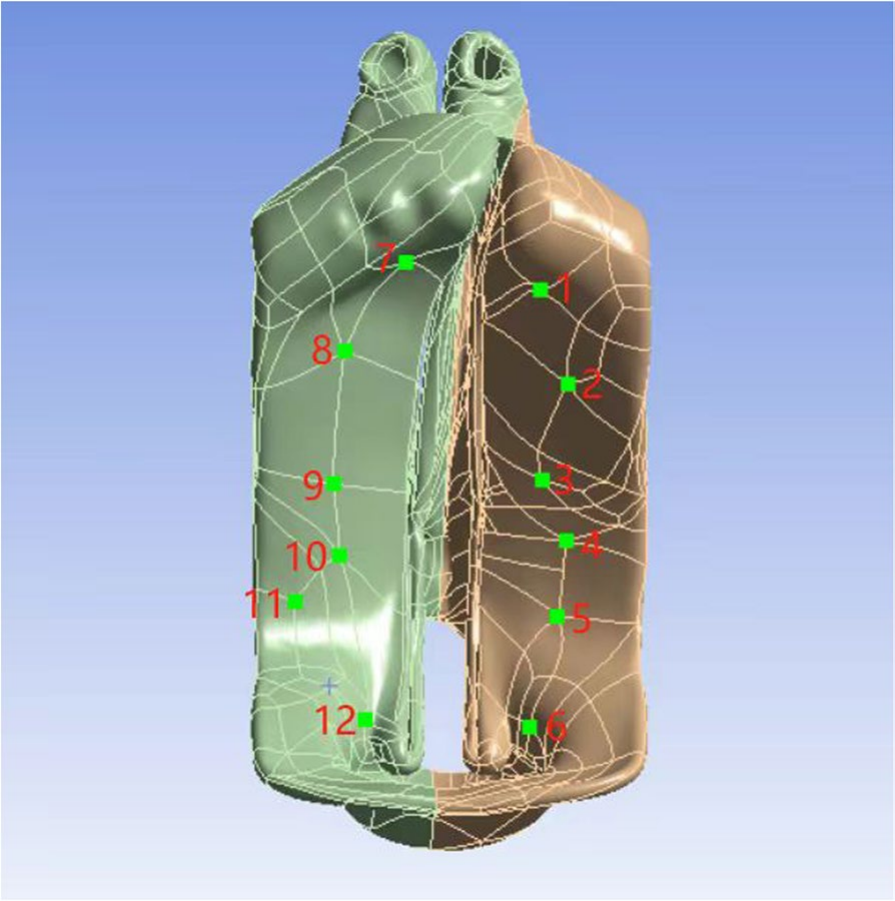
Diagram of the outer points of the suture

**Fig. 4:**
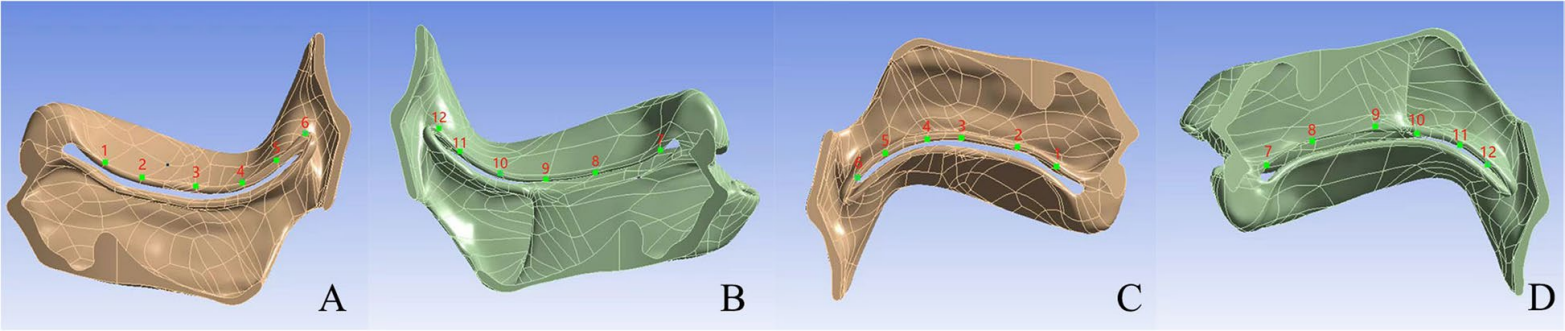
**A** Schematic diagram of surgical points on the right site of oral surface. **B** Schematic diagram of surgical points on the left side of oral surface. **C** Schematic diagram of the right point of nasal surface operation. **D** Schematic diagram of the left point of nasal surface operation

As detailed in Fig. 3 and Table 1, the highest tension is observed at Node 3 (0.32388 MPa, 0.40681 MPa/mm) along the right suture and at Node 12 (0.4379 MPa, 0.21244 MPa/mm) along the same suture. In the oral facial operation flaps, Node 1 on the right experiences the highest tension (0.93218 MPa, 0.29438 MPa/mm), while on the left, it is Node 10 (0.29217 MPa, 0.08103 MPa/mm, Fig. 4A, B, Table 2). In the nasal operation flaps, Node 6 on the right has the highest tension (0.080944 MPa, 0.04797 MPa/mm), and on the left, it is Node 7 (0.38239 MPa, 0.25901 MPa/mm) (Fig. 4C, D, Table 3).

## Discussion

**Fig. 5:**
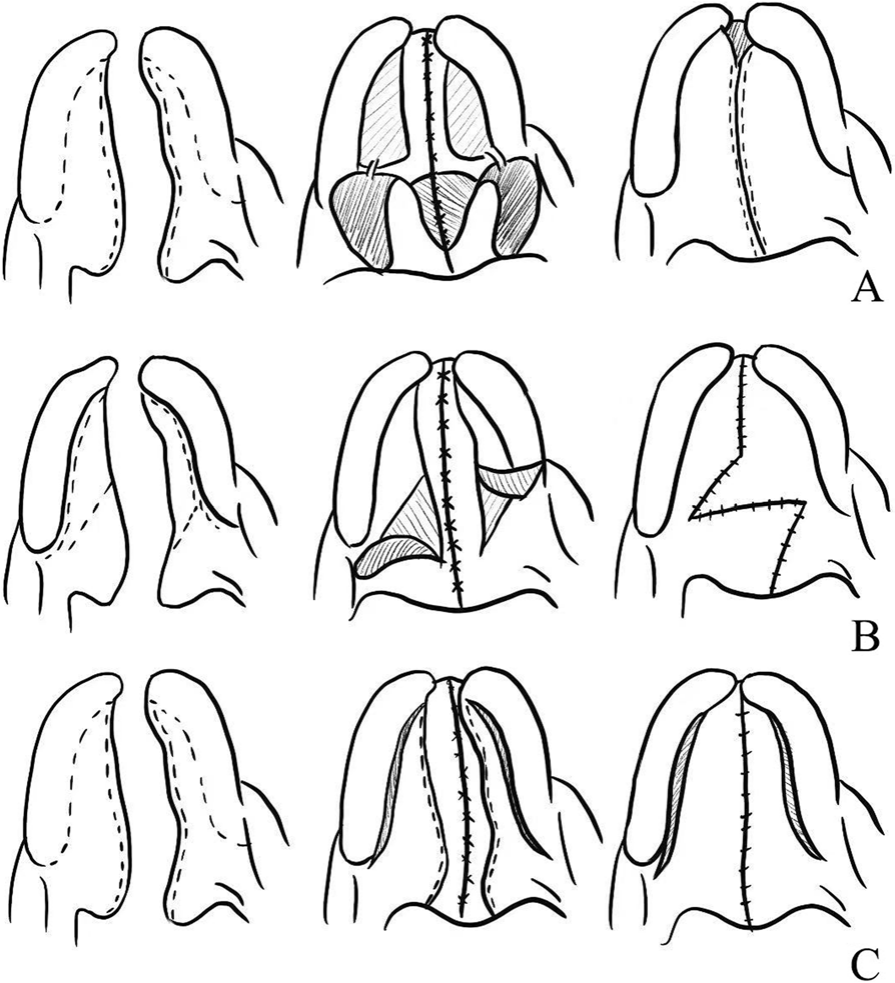
Major surgical procedures for cleft lip and palate. **A** Illustration of two-flap palatoplasty technique. **B** Illustration of Furlow palatoplasty. **C** Illustration of Von Langenbeck palatoplasty

In this study, we developed a three-dimensional finite element model of a cleft palate to analyze deformation and tension at common surgical incision sites used in reconstruction surgery. Our findings reveal critical insights into tension and deformation patterns, particularly identifying several key points along the suture on both the oral and nasal surfaces that exhibit the highest levels of tension and deformation. Reference values are applied by taking into account values defined in previous studies. The biomechanical properties of human skin are complex, and characterized by nonlinearity, viscoelasticity, and anisotropy, among others [23]. The mechanical properties of linear elastic materials are often characterized by measuring Young’s modulus [25]. A finite element model of unilateral cleft lip nasal deformity was conducted by Huang and his team, based on MRI (magnetic resonance imaging) data from one volunteer, including several cartilage frameworks and a skin envelope [24]. The direction of loading points and forces in the study was also selected according to experience, and the peripheral nodes of the model were set as fixed nodes. This Narrows down the possible range of surgical options. Akdemir et al. evaluated the maxillofacial stress distribution in patients with unilateral cleft lip and palate after different maxillary advancement schemes [26]. The results of this study provide information on the initial stress distribution and displacement patterns during maxillary extension in patients with cleft lip and palate, but because the soft tissue and postoperative scar tissue of cleft lip and palate were not considered in the modeling process, the actual treatment results may be different. However, our model provides a more nuanced understanding of tension distribution, indicating that this area is particularly susceptible to increased stress during surgery. This insight can inform surgical decisions, suggesting that greater care should be taken when operating in high-tension regions to minimize complications. When comparing our results with previous research, we note that traditional surgical techniques, such as Von Langenbeck palatoplasty, involve incisions made parallel to the fissure and gingiva (see Fig. 5) [7, 26]. Our findings align with earlier studies that highlight tension at the medial root of the cleft palate as a significant concern.

Our analysis of the two-flap palatoplasty technique, which involves creating two flaps along the suture and stitching them together (see Fig. 5A) [10], suggests a potential improvement in surgical outcomes. Our findings can guide surgeons to identify a more optimal fold-over point at the middle suture, where deformation values are relatively lower, thereby potentially reducing the risk of postoperative complications.

Furlow palatoplasty, which utilizes two relaxed incisions to mitigate high incision tension (see Fig. 5B) is supported by our findings. The deformation data indicate that this technique may significantly enhance healing outcomes by minimizing stress at critical points during recovery. By highlighting these techniques and their implications for surgical practice, our study contributes valuable insights that could inform clinical strategies for cleft palate repair.

The use of 3D finite element analysis in cleft palate surgery offers several distinct advantages. This method allows for the simulation of complex mechanical behaviors, enabling visualization of stress distributions in multiple dimensions and more accurate predictions of surgical outcomes. Compared to traditional two-dimensional models, our 3D approach provides a comprehensive understanding of how various surgical techniques impact the palate’s biomechanical properties.

However, previous research in 3D modeling for cleft palate surgery has faced limitations, such as oversimplified assumptions regarding material properties or inadequate representation of tissue interactions. However, parameters such as cartilage size and skin depth are challenging to obtain in practice and vary significantly across a large cohort. Moreover, their use leads to extensive computation, delaying the production of patient models and the formulation of operation management strategies.

Furthermore, the 12 key points in our study determined based on potential surgical incisions, are not fixed and could vary, leading to some inevitable deviations in results. While the finite element model is a valuable tool, key factors such as nonlinear tissue properties, patient-specific anatomical differences, and long-term effects of surgical intervention should be adequately considered.

Addressing these limitations, as well as future directions like the use of more advanced material properties or clinical validation, would provide a more balanced assessment of your model's clinical applicability. We anticipate that future advancements in material properties and the integration of artificial intelligence will help overcome these challenges.

## Conclusions

In summary, we developed a 3D finite element model of the cleft palate using computer technology, which facilitates mechanical analysis and simulation of deformation and tension across various parts of the cleft palate incision under realistic conditions. This model provides insights into the mechanical impacts on the oral and nasal cavities. We identified regions of maximum deformation and tension through analysis of 12 key points selected during the simulation of the suture, oral, and nasal surfaces. These data can guide surgeons in determining the optimal location, direction, size, and suturing technique for surgical incisions. Additionally, this technology allows patients with different types of cleft lip and palate to customize their own models, aiding doctors in devising the best surgical plans. The ultimate goals are to enhance wound healing, improve postoperative outcomes, reduce complications, and hopefully better the prognosis for patients with cleft lip and palate at various levels.

## Data Availability

All data generated or analyzed during this study are included in this published article.

## Abbreviations

CT: Computed tomography
PLY: Polygon file format
STP: Standard for the exchange of product data
MRI: Magnetic resonance imaging
MPa: Megapascal
